# Modern Methods of Producing Local Anesthesia

**Published:** 1912-03-15

**Authors:** Herman Prinz

**Affiliations:** St. Louis, Mo.


### MODERN METHODS OF PRODUCING LOCAL ANESTHESIA.

BY HERMAN PRINZ, M.D., D.D.S., ST. LOUIS, MO.

*Means of Producing Local Anesthesia.* The term anesthesia (without sensation), which was suggested in 1846 by the great physician-litterateur, Oliver Wendell Holmes, to Dr. Morton, is usually defined as an artificial deprivation of all sense of sensation, while the mere absence of pain is referred to as analgesia. Correctly speaking, the term local anesthesia is partially a misnomer. In producing local anesthesia we do not fully comply with all the requirements that anesthesia demands, because a part of the sensorium —the sense of touch, for instance—is not abolished. The term local anesthesia has, however, acquired such universal recognition that it would seem unwise to recommend a change.

Anesthesia may be artificially produced by inhibiting the sensory nerve fibers at their central end-organs in the brain or at their peripheral end-organs in the tissues, thus producing general and local anesthesia. Local anesthesia may be obtained in two definite ways. We may inhibit the function of the peripheral nerves in a circumscribed area of tissue, and we refer to this process as "terminal anesthesia," while, if we block the conductivity of a sensory nerve trunk somewhere between the brain and the periphery, we speak of it as "conductive anesthesia." Conductive anesthesia may be produced by injecting into the nerve trunk proper—endoneural injection—or by injecting into the tissues surrounding a nerve trunk—perineural injection. The latter form is the usual method pursued when conductive anesthesia for dental purposes is indicated. Specific forms of local anesthesia may also be produced by paralyzing the sensory ganglia in the brain or in the spinal cord; these methods have, however, no bearing on the subject under consideration.

The successful practice of local anesthesia involves the carefully adjusted co-operation of a number of important details, each one constituting a definite factor in itself, which, when neglected, must necessarily result in failure. As a whole, the practice of local anesthesia by the hypodermic method represents the composite of the following factors:

1. A solution of active ingredients corresponding to the physical and physiologic laws which govern certain functions of the living cell.
2. A carefully selected hypodermic armamentarium.
3. A complete mastery of the technique.
4. A proper selection of the correct method suitable for the case on hand.
5. Good judgment of prevailing conditions.

*Physiologic Action of Anesthetics.* According to more recent therapeutic conceptions, it is generally recognized that a drug or combination of drugs which simultaneously produce local anemia and inhibition of the sensory nerves in a circumscribed area of tissue is the logical solution of the question of local anesthesia. Certain important factors, however, relative to the physiologic and physical action of the solution employed for hypodermic injection upon the cell govern the successful application of such methods. It is of prime importance, therefore, to comply with the laws regulating the absorption of injected solutions—osmotic pressure.

If we separate two solutions of salt of different concentration by a permeable animal membrane, a continuous current of salt and water results, which ceases only after equalization of the density of the two liquids—that is, equal osmotic pressure (according to the Boyle-Vant Hoff law) is established. The current passes in both directions, drawing salt from the stronger to the weaker solution, and water vice versa, until osmotic equilibrium is obtained. The resultant solutions are termed according to De Vries, isotonic.

Osmotic pressure is a physical phenomenon possessed by water and all aqueous solutions, and is dependent on the number of molecules of salt present in the solution and on their power of dissociation. In organized nature these osmotic interchanges play an important role in regulating the tissue fluids of both animals and plants. In the animal tissue the ciculation depends principally upon the mechanical force exerted by the heart. The life of the cell depends on the continuous passage of these fluids, which furnish the nutrient materials, consisting of water, salt, and albumin. These chemicals are normally present in certain definite proportions. The membrane of the living cell is, however, only semi-permeable—that is, the cell readily absorbs distilled water when surrounded by it. The cell becomes macerated, loses its normal structure, and finally dies. If, on the other hand, the surrounding fluid be a highly concentrated salt solution, the solution absorbs water from the cell; no salt molecules enter into the cell body proper. The cell shrinks, and finally dies. This process of cell death is in general pathology referred to as necrobiosis. Another important factor teaches that all aqueous solutions that are isotonic possess the same freezing point—that is, all solutions possessing an equal freezing point are equi-molecular, and possess equal osmotic pressure. This law of physical chemistry has materially simplified the preparation of such solutions. The freezing point of human blood, lymph, serum, etc., has been found to equal approximately 0.55 degrees C., which in turn corresponds to a 0.9 per cent. sodium chlorid solution. Such a solution is termed a physiologic salt solution. In the older works on physiology a 0.6 per cent. sodium chlorid solution is referred to as a physiologic salt solution, and corresponds to the density of the blood of the frog. A slight deviation above and below the normal percentage of the solid constituents is permissible. When physiologic salt solution at body temperature is injected into the loose connective tissues under the skin in moderate quantities, neither swelling nor shrinking of the cell occurs. A simple wheal is formed, which soon disappears, and, as no irritation results, consequently no appreciable pain is felt. Other similar bodies that are equally soluble in water act in the same manner, with the exception of the salts of the alkali and alkaline earth metals—as potassium or sodium bromid. The latter substances produce intense physical irritation, followed, however, by prolonged anesthesia, and in the consequence are termed by Liebreich painful anesthetics. If, on the other hand, simple distilled water is injected, only a superficial anesthesia is produced; the injection itself is very painful, and acts as a direct protoplasm poison by maceration of the cell contents, which results in the death of the cell. If distilled water approximately at a ratio of ten drams to the pound of body weights is injected into dogs, they will succumb in a short time. The injection of higher concentrated salt solutions produces opposite effects; water is removed from the tissues with more or less pronounced pain, and followed by superficial anesthesia. The red blood corpuscles are extremely susceptible to any injected fluid which is not isotonic in its nature. They are universally destroyed (hemolysis) by the injection of fluids which are not represented by an isotonic salt solution. Severe tissue disturbances result, which may terminate in gangrene. Hypotonic solutions—solutions containing less than 0.9 per cent. of sodium chlorid—cause swelling of the tissue, while hypertonic solutions—solutions containing more than 0.9 per cent. of sodium chlorid—produce shrinkage. These manifestations are proportionately the more intense the further the solution is removed from the freezing point of the blood. Furthermore, hypotonic as well as hypertonic solutions require much more time for their absorption than isotonic solutions, as the osmotic pressure has to be standardized to the surrounding fluids—that is, to the isotonic index of the tissue fluids. Local anemia, or ischemia—a temporary constriction of circulation—prevents, as it has been experimentally shown, the rapid absorption of fluids that are injected into the affected area. Retarded absorption of the injected fluid, holding poisonous drugs in solution, means increased action of these poisonous drugs within the injected area. Increased action denotes increased consumption of the poisonous drugs, and, as a consequence, there is less danger from general absorption. The more important means applied for the purpose of producing local anemia are:

1. The Esmarch elastic bandage.
2. The application of cold.
3. The extract of the suprarenal capsule, or its synthetic substitutes.

Some observers have maintained that local anemia produces anesthesia. This is not, however, the case, as it is merely an important means to confine the injected anesthetic to the anemic region, and thus bring about an increased and prolonged action of the drug. Consequently the concentration of the anesthetic solution may be of a lower percentage, which, of course, lessens the danger of intoxication. For obvious reasons the Esmarch elastic bandage can not be made use of for dental operations.

Physically reducing the temperature of the body by the application of cold (ice pack, ice and salt mixture, cold metals, etc.) was practiced by the older surgeons. James Arnott, in 1848, suggested the adoption of deminished temperature as "a safer mode than any hitherto in use of producing insensibility in surgical operations," and Blundell, in 1855, advocated ice packs and salt solutions as means of producing "local anesthesia by congelation" for dental purposes. Through the efforts of Sir B. W. Richardson, in 1866, this method was placed on a rational basis by the introduction of his ether spray. To obtain good results, a pure ether (boiling point 95° F., 35° C.), free from water, is necessary. Certain other hydrocarbons possess similar properties in varying degrees, depending on their individual boiling point. In 1867 Rottenstein called attention to the use of ethyl chlorid as a refrigerating agent, and Rhein, in 1889, introduced methyl chlorid for the same purpose. In 1891 Redard reintroduced ethyl chlorid as a local anesthetic, which since has become known by many trade names, as antidolorine, kelene, narcotile, etc.—and mixtures of the first two in various proportions, known as anestol, anestile, coryl, methethyl, etc., are extensively used in minor oral and general surgery. A pure ethyl chlorid (boiling point 55° F., 13 °C.) is best suited for this purpose, as it lowers the temperature of the tissues sufficiently to produce a short superficial anesthesia in a few minutes. Too rapid cooling or prolonged freezing by methyl chlorid (boiling point 12° F., 24.5° C.), or the various mixtures thereof, produce deeper anesthesia, but such procedures are dangerous. They frequently cut off circulation in the affected parts so completely as to produce sloughing (necrosis). Liquid nitrous oxid gas, liquid or solid carbonic acid (recently known as carbonic acid snow), and liquid air, all of which have a boiling point far below zero, are recommended for similar purposes, but they require cumbersome apparatus and are extremely dangerous.

*Ethyl Chlorid and its Administration.* Ethyl Chlorid—Monochlorethane; hydrochloric ether, C2 H5 Cl. "A haloid derivative, prepared by the action of hydrochloric acid gas on absolute alcohol." At normal temperature, ethyl chlorid is a gas, and under a pressure of two atmospheres it condenses to a colorless, mobile, very volatile liquid, having a characteristic, rather agreeable, odor and burning taste. It boils at about 55° F., (13° C.) and is very inflammable, burning with a smoky, green-edged flame. It is stored in sealed glass or metal tubes, and when liberated at ordinary room temperature (70° F., 21° C.) it evaporates at once. In commerce it is supplied in plain or graduated glass tubes of from 3 to 60 grams capacity, or stored in metallic cylinders holding from 60 to 100 grams or more. To remove the ethyl chlorid from the hermetically sealed smaller tubes, the neck has to be broken off, while the larger glass and metallic tubes are provided with suitable stop cocks of various designs to allow definite amounts of the liquid to be released.

*Mode of Application.* For the extraction of teeth, immediate removal of the pulp, opening of abscesses, and other minor operations about the oral cavity, the tube should be warmed to body temperature by placing it in heated water, and its capillary end should be held about six to ten inches from the field of operations. The distance depends on the size of the orifice of the nozzle, and complete vaporization should always be produced. The Gebauer tube is fitted with a spray nozzle, which shortens the distance to one or two inches, and is especially well adapted for dental purposes. The stream is directed upon the tissues until the latter are covered with ice crystals and have turned white. For the extraction of teeth the liquid should be projected directly upon the surface of the gum, as near the apex of the root as possible, but care should be taken to protect the crown of the tooth on account of the painful action of cold on this part. Tissues to be anesthetized should first be dried and well surrounded by a film of vaselin or glycerin, and protected by cotton rolls and napkins, to prevent the liquid from running into the throat. Let the patient breathe through the nose. Occasionally light forms of general anesthesia are induced by inhaling vapor. On account of the difficulty of directing the stream of ethyl chlorid upon the tissues in the posterior part of the mouth, it is not successfully applied in those regions. The intense pain produced by the extreme cold prohibits its use in pulpitis and acute pericementitis. To anesthetize the second and third branch of the fifth nerve, it is recommended to direct the stream of ethyl chlorid upon the cheek in front of the tragus of the ear, but the author has not seen good results from such a procedure. Caution should be exercised in using ethyl chlorid near an open flame or in conjunction with the thermo-cautery, as severe burns have resulted by setting the inflammable vapor on fire.

*Adenalin.* Within the last decade the active principle of the suprarenal capsule has evoked extensive comments in therapeutic literature. It has been isolated by a number of investigators under different names, as epinephrin by Abel (1897) suprarenin by Fuerth (1898), and adrenalin by Takamine and Aldrich (1901). Many other titles are given to this chemical—as adnephrim, adrin, paranephrin, suprarenalin, supracapsulin, hemostasin, etc. The United States Pharcopoeia (eighth revision) has not as yet admitted this alkaloid to its pages, and, therefore, whenever we refer here to the hydrochloric salt of the alkaloid of the suprarenal capsule, we speak of it as adrenalin, the term which is at present preferred in the United States. Adrenalin is a grayish-white powder, slightly alkaline in reaction, and perfectly stable in dry form. It is sparingly soluble in cold and more soluble in hot water, is insoluble in ether or alcohol, and with acids it readily forms soluble salts. The preparation that is employed mostly for therapeutic purposes is a solution of adrenalin hydrochloride in a 1 to 1000 physiologic salt solution, to which preservatives—as small quantities of chloretone, thymol, etc.—are added. Adrenalin solution does not keep well. On exposure to air and especially in the presence of even minute quantities of an alkali it is easily oxidized, becoming pink, then red, and finally brown, and with this change of color its physiologic property is destroyed. If the adrenalin solution be further diluted, it becomes practically worthless within a few days.

When adrenalin is injected into the tissues, even in extremely small doses, it temporarily raises the arterial blood pressure, acting as a powerful vasoconstrictor by stimulating the smooth muscular coat of the blood vessels, and thus produces local anemia. Large doses finally reduce the blood pressure, and heart failure results. The respiration at first quickly increases, but slows down and finally stops with expiration. Its action is largely confined to the smooth muscle fibers of the peripheral vessels. Adrenalin is destroyed by the living tissue cells, the body ridding itself of the poison in some unknown manner. While adrenalin does not possess local anesthetic action, it increases very markedly the effect of certain anesthetics when combined with them. Very recently, it has been shown by Esch, that adrenalin possesses a specific action on nerve tissue, viz: it prepares the latter tissue in a peculiar way so as to take up the anesthetic more readily. Esch compares this action with the use of a mordant in the dyeing industry, viz.: to "fix" the color. These observations are of vast importance in connection with the production of local anesthesia. Carpenter, Peters, Moller, and others referred to the use of adrenalin in this respect, and finally Braun, in 1902, published his classic researches, and to him and his co-workers, especially Heinze and Lawen, belong the credit of establishing a rational basis for the production of local anesthesia. It is claimed that secondary hemorrhage frequently occurs after the anemia produced by the adrenalin has subsided, and that the tissues themselves suffer from the poisoning effect of the drugs, resulting in necrosis. Such results are produced only by the injection of too large quantities of the drug, which by their deeper action close up the larger arteries. The prolonged anemia will give way to a dilatation of the blood vessels, and, if the tissues are too long deprived of the circulation, we are able to understand why sloughing may result. Small doses of adrenalin have no effect upon the tissues or on the healing of a wound. Palpitation of the heart and muscular tremor, which were occasionally noticed in the early period of the use of the drug, are the direct result of too large doses. Recently a synthetic adrenalin has been successfully prepared by Stolz, which, with hydrochloric acid, forms a stable and readily soluble salt. It is known as synthetic suprarenin hydrochlorid. The new chemical has been carefully tested physiologically and in clinical work, and the general consensus of opinion points to the fact that it is not alone equal, but in certain respects superior, to the organo-preparations. Synthetic suprarenin solutions may be readily sterilized by boiling. They are relatively stable, and their chemic purity insures uniform results. They are comparatively free from dangerous side actions. The writer's observations regarding the value of synthetic suprarenin relative to its actions and its general behavior is in full accordance with the above statements, and its advantages over the organo-preparations has led us to adopt it as a component in the preparation of local anesthetic solutions. For dental purposes—that is, for injecting into the gum tissue—the dose may be limited to one drop of the adrenalin solution (1 to 1000) or the synthetic suprarenin solution (1 to 1000), added to each cubic centimeter of the anesthetic solution, 5 drops being approximately the maximum dose to be injected at one time.

*Novocaine.* Ever since the introduction of cocain for the purpose of producing local anesthesia, quite a number of substitutes have been placed before the profession, for which superiority in one respect or another is claimed over the original cocain. The more prominent members of this group are tropacocaine, the eucaines, acoin, nirvanin, alypin, stovaine, novocaine, and very recently, quinin and urea hydrochlorid. None of these compounds, with the exception of novocaine, has proved satisfactory for the purpose in view. The classical researches of Braun have established certain factors which are imperative to the value of a local anesthetic. These factors concern their relationship to the tissues in regard to their toxicity, irritation, solubility and penetration, and to the toleration of adrenalin.

There is no need at this moment to enter into a discussion of the pharmacologic action of the drugs usually classified as local anesthetics. Let it suffice to state how the above-mentioned drugs fulfill the demands of Braun. Tropacocaine is less poisonous, but also less active than cocain, it completely destroys the action of adrenalin; the eucaines partially destroy the adrenalin action, they are, comparatively speaking, equally as poisonous as cocain; acoin is irritating to the tissues and more poisonous than cocain; nirvanin possesses little anesthetic value; alypin and stovaine are closely related, producing severe pain when injected, which occasionally has resulted in necrosis. Quinin and urea hydrochlorid reacts strongly acid and, as a consequence, severely damages the tissues in the injected area. As we have recently shown elsewhere it possesses no advantage when employed as a local anesthetic in dental operations but has many disadvantages as compared to cocain or novocaine.

Novocaine alone fully corresponds to every one of the above claims. Its toxicity is about two to six times less than cocain; it does not irritate in the slightest degree when injected, consequently no pain is felt from its injection per se; it is soluble in its own weight of water; it will combine with adrenalin in any proportion without interfering with the physiological action of the latter, and it will be readily absorbed by the mucous membrane. The studies of Biberfield and Braun brought to light another extremely interesting factor concerning the novocaine-adrenalin combination. Both experimenters working independently of each other, observed that the adrenalin anemia on the one hand, and the novocaine anesthesia on the other hand were markedly increased in their total effects upon the tissues. Consequently, a smaller quantity of this most happy combination is required to produce the same therapeutic effect as a large dose of each individual drug alone would produce when injected separately. The injection of a solution of the combined drugs is precisely confined to the injected area, general effects are therefor rarely produced.

Novocaine is the hydrochloric salt of a synthetically prepared alkaloid, the methyl ester of p-aminobenzoic acid. It is a white crystalline powder, or colorless needle-shaped crystals, melting at 263° F. (156° C.). It may be heated to 200° F. (120° C.) without decomposition. It dissolves in an equal amount of cold water, the solution having a neutral character; in cold alcohol it dissolves in the proportion of 1 to 30. Caustic alkalies and alkaline carbonates precipitate the free base from the aqueous solution in the form of a colorless oil, which soon solidifies. It is incompatible with the alkalies and alkaline carbonates, with picric acid, and the iodides. Its solutions may be sterilized by boiling without decomposition.

As stated above, the relative toxicity of a given quantity of cocain in solution depends upon its concentration; this same peculiarity is not shared by novocaine. The dose of novocaine may be safely fixed at one-third of a grain for a single injection. For dental purposes a 1½ or a 2 per cent. solution in combination with adrenalin has been injected without any ill results. For the purpose of confining the injected novocaine to a given area, the addition of adrenalin in small doses, on account of its powerful vasoconstrictor action, is well adapted. It is the important factor which prevents the ready absorption of both drugs and largely nullifies poisonous results.

An injection of two drops of a ten per cent. solution of novocaine produces a diffuse anesthesia lasting approximately twenty minutes; the same quantity with the addition of one drop of adrenalin solution increases the anesthetic period to one hour and also localizes the effect in the injected area.

A suitable formula may be made as follows:

| | |
|---|---|
| ℞ Novocaine | 10 grains |
| Sodium Chloride | 4 grains |
| Distilled Water | 1 ounce. |
| Mix and boil. | |

To each hypodermic syringe full add two drops of the adrenalin solution when used.

For convenience the formula has been compounded and put on sale in tablet form containing: Novocaine, one third of a grain; Synthetic suprarenin hydrochlorid, one twelve-hundredth of a grain, and sodium chloride one-third of a grain. One such tablet dissolved in 20 drops of sterile water makes a two per cent. solution.

Solutions are in this way easily made when needed by dissolving a tablet in the water.—*Dental Summary*, February, 1912.

